# All P's or mixed vegetables?

**DOI:** 10.3389/fpsyg.2013.00234

**Published:** 2013-05-08

**Authors:** Michael K. Tanenhaus

**Affiliations:** Brain and Cognitive Sciences, University of RochesterRochester, NY, USA

In a beautifully written, cogently argued paper MacDonald (2013), presents the theoretical framework that guides one of the most creative and influential research programs in the language sciences. The PDC began with empirical demonstrations that readers are remarkably sensitive to distributional patterns in the input. These empirical demonstrations were accompanied by theoretical arguments that ambiguity resolution can be modeled by constraint-based (probabilistic) systems that learn these patterns from experience (also see Tanenhaus and Trueswell, 1995; Tabor et al., 1997). This research was part of a wave of research in the 1990's that answered long-standing questions in real-time language comprehension and early language acquisition, with variations of “It's the input, stupid” (e.g., Saffran, Aslin, and Newport's seminal work on statistical learning, Saffran et al., 1996).

The probabilistic constraints that comprehenders learn and use *must* arise from the output created by speakers and writers. But why does that output exhibit systematic patterns both within and across language? One answer is that languages maximize learnability for the child. A second is that speaking and more generally the structure of language is shaped by considerations of communicative efficiency (see Jaeger's commentary). MacDonald proposes a production-based answer. Grounding her arguments in insights from the motor planning and control literature, MacDonald makes a convincing case that that constraints on planning processes in language production play an important role in shaping the form of utterances. She argues that the demands of memory retrieval, planning, and linearization for sequential behavior that requires a hierarchical control structure play the central role both in determining the forms that speakers use to convey their intentions and, as a consequence, the patterns of linguistic forms that are observed both within and across languages. These arguments are supported by a clear exposition of principles and a summary of some elegant experiments focusing on the production of relative clauses.

Less convincing is MacDonald's argument that these production constraints comprise *most* of the story. Here is the cartoon view of the assumptions that underlie this claim. Speaking is extremely hard while understanding is comparatively easy. It is costly for speakers to take into account the listener, especially at the temporal grain required for influencing the planning process in production. Listeners, however, are really good at learning probabilistic constraints, making use of context, and adapting to speakers. Given these considerations it makes sense for speakers (and languages) to promote forms that make speaking easier.

In this commentary, I raise question about some of the assumption that underlie the claim that production demands—in particular planning and linearization—are most of the story. I begin by noting some parallels with earlier arguments that were based on assumptions about the difficulty of comprehension. I note that much of what we know about how naturally listeners use context, emerged only when psycholinguists began to examine language comprehension in richer contexts and more natural interactive tasks. I suggest that we actually don't know much about how speakers might adapt to addressees in real-time language production. There is a paucity of research that examines production in those interactive environments where addressees provide feedback. When production is examined in interactive settings, there is tantalizing evidence that speakers do, in fact, monitor addressees, and might adapt on the fly.

## Is speaking is much harder than understanding?

A conservative answer is that we really don't know. The answer likely depends on the metric used to quantify and compare difficulty. When we examine the task that the listener faces, it seems daunting. The listener must infer speaker intentions from a transient series of acoustic events. By analogy, imagine reading text without spaces as it passes through a two-letter aperture at a variable rate which you do not control, and with some of the features of the letters arriving asynchronously. In fact, after Marslen-Wilson's classic, and at the time surprising, studies demonstrating the remarkable speed of real time spoken language comprehension (Marslen-Wilson, 1973, 1975), psycholinguists wondered how such a complex task could be performed so rapidly^1^.

My point is that we often make assumptions that a (seemingly) complex process is hard without independent motivation. To further illustrate this point, psycholinguists have assumed, and many still do, that listeners do not take the perspective of the speaker into account in real-time processing because doing so is too resource demanding (e.g., Keysar et al., 2000). Moreover, pragmatic inferences are too slow and too costly to influence syntactic processing (Clifton and Ferreira, 1989). Central to these arguments is the intuition that pragmatic and similar processes are strategic, and that we know that strategic processes are slow and resource demanding (Posner and Snyder, 1975; Shiffrin and Schneider, 1977).

## Rich context and natural tasks

In studies of real-time comprehension in more natural tasks, including interactive conversation, listeners are remarkably adept at rapidly doing things that we previously assumed were slow and costly. These include constructing referential domains on the fly, taking into account action-based affordances, computing implicatures, and modifying referential domains based on the speaker's knowledge and perceived intentions. In fact, information provided by specific context can override even strong expectations based on distributional patterns that arise from accumulated experience (for review of some of this early work see Tanenhaus and Brown-Schmidt, 2008). Space limits preclude developing this argument, so I will simply make the claim that many demonstrations that listeners are egocentric, inferences are costly, etc., fall by the wayside when we begin to understand how listeners (and speakers) construct and restrict domains (for discussion see Degen and Tanenhaus, under review).

## Do speaker's monitor addressees during utterances planning and production?

MacDonald suggests that whereas listeners might be good at using context, adapting to speakers, etc., speakers find this costly and therefore do not take the listener into account, at least at a fine-enough temporal grain to influence planning. Do speakers take into account real-time feedback from addressees and adapt on the fly? The received view in the production literature is that speakers do not. But, I would argue that we really don't know the answer because most production research does not use interlocutors in natural tasks. One reason why listeners might be so good at adapting to speakers is that listeners can use an internal model to generate expectations that create an error signal as the speaker-generated input is processed. This error signal can drive learning accumulated over experience and also rapid adaptation (see Jaeger's commentary). In production, however, the speaker can only adapt to the listener if she provides an error signal that the speaker can use.

Try this exercise. In a conversation with an interlocutor, avoid giving the speaker any feedback; don't raise your eyebrows, nod or say, “uh-huh,” “hmm,” “ok,” and “really.” More often than not the speaker will become hesitant and her speech will become halting and disfluent. Why? One plausible explanation is that speakers monitor addressees for this kind of feedback. Consider another example, which my colleague, James Allen, uses to argue for incremental generation systems which incorporate feedback. You begin to ask your partner to “Hand me the Phillips tool… ” which is in a pile with some screwdrivers. Noticing that he is confused, you might change your utterance, to something more descriptive (e.g., “it looks like, uh, a screwdriver with notches at the end… ”) and subsequently use descriptive terms for less common tools. Most production experiments, however, do not create the opportunity for feedback. In the few experiments where feedback is available (Snedeker and Trueswell, 2003; Clark and Krych, 2004; Roche et al., under review) speakers do in fact adjust their utterances (For other examples, see Brennan and Hanna, 2009).

A different type of evidence that speakers might take into account the perspective of their addressees during utterance planning comes from recent research on use of referring expressions. Wu and Keysar (2007) introduced a paradigm in which pairs of naïve participants together learn some novel names for novel shapes (Wu and Keysar, 2007). One partner, who is subsequently the director in a referential communication task, then learns some new (privileged) names. When the director instructs the matcher to click on a target shape, the simple, name-alone form is only used for shared names. For privileged names, speakers use descriptions, which are far more complex (Heller et al., 2012; Gorman et al., 2013). When directors do use a name for a privileged shape, two things happen: the name is immediately followed by a description and the name is realized prosodically in such a way the listeners can reliably tell that further information is following the name (compared to productions of shared names). If we assume absence of perspective taking in production, this is a surprising result. Perhaps this is the exception that proves the rule because shared experience makes the speaker-specific information immediately available when a name is retrieved. This is certainly a possibility. However, another possibility is that speakers actively tailor aspects of their utterances to convey or assess likely common ground. The following exercise that suggests that speakers are sensitive to at least some types of feedback.

This exercise requires you and two other participants, all of whom know that one of the participants is an expert on Topic A. Speakers generally ask information questions when they don't already know the answer, and they direct those questions to people who they expect to have the relevant knowledge (indeed listeners rapidly use this information to disambiguate potential referents, Brown-Schmidt et al., 2008). Address a question about the expert's bailiwick to the person with less expertise and observe the reaction of both parties. Examples like this raise the possibility that speakers sometimes provide specific information in a bid to assess a listener's likely knowledge, and to correct an interlocutor who isn't appropriately calibrated. In addition, interlocutors may mark and track who can be taken as the source of information. If A tells B that Harry is in the hospital, B might answer, “Oh, really,” but not “Yes, really.” “Yes,” conveys independent knowledge, whereas “Oh,” does not (Gunlogson, 2008). We don't know that speakers track this information and if they do, how it might affect utterance planning. However, the fact that interactive conversation contains many linguistic devices that signal knowledge state and intention suggests that examining these phenomena might change our ideas about what information sources speakers attend to in much the same way that our perspective on language understanding has been affected by similar experiments.

## Conclusion

In conclusion, I am not challenging MacDonald's persuasive arguments that the form of utterances, and language itself is strongly influenced by memory and planning constraints. Neither am I challenging the claim that production might be harder than comprehension, nor suggesting that incorporation of real-time feedback plays the same role in production as it does in comprehension. What I am arguing, however, is that, as was the case for language comprehension, we won't know until we ask the relevant questions in interactive environments that provide feedback that speakers might or might not use. Otherwise, we are likely to underestimate what speakers can do on the fly, much as we once underestimated what listeners can do.

## Footnotes

^1^The relentless and, in my opinion, often misguided focus on what happens during the earliest moments in language processing is a reflex of the assumption that one-line spoken language processing is really hard (Tanenhaus, 2004). Proposals about information encapsulation were motivated, in part, by the need to explain how word recognition and parsing could occur so rapidly (Fodor, 1983). Moreover, the demands of on-line processing motivated proposals that languages follow principles that minimize the real-time parsing difficulty (Hawkins, 1990, 1995).
